# The Orexin System Modulates Stress‐Induced Alcohol Preference and Reinstatement in Adolescents: Bioinformatics and Experimental Evidence

**DOI:** 10.1111/adb.70173

**Published:** 2026-06-23

**Authors:** Wenhao He, Aqian Hu, Tianshu Zhao, Li Deng, Huilin Wu, Xiaojun Xiang

**Affiliations:** ^1^ Department of Psychiatry, National Clinical Research Center for Mental Disorders, and National Center for Mental Disorders The Second Xiangya Hospital of Central South University Changsha Hunan China; ^2^ Affiliated Mental Health Center & Hangzhou Seventh People's Hospital, School of Brain Science and Brain Medicine Zhejiang University School of Medicine Zhejiang Hangzhou China

**Keywords:** alcohol preference and reinstatement, animal experiments, orexin system, protein–protein interaction network, stress

## Abstract

Underage drinking has become a global public health concern. One of the major causes of underage drinking is stress. The orexin system has been reported to be involved in both alcohol addiction and stress. However, few studies have examined this system, especially among adolescents. Therefore, we constructed protein–protein interaction (PPI) networks to confirm that orexin receptors are connected to stress‐ and alcohol dependence‐related genes, providing a theoretical basis for our experimental approach. Animal experiments employed the conditioned place preference (CPP), the foot‐shock stress model and the enzyme‐linked immunosorbent assay (ELISA), to elucidate the role of the orexin system in the stress‐induced alcohol addiction‐related behaviour among adolescent mice. Our results revealed that there were interactions among orexin system, chronic/acute stress and alcohol dependence related proteins. Otherwise, chronic stress can increase the animals' vulnerability to alcohol addition‐related behaviour. Additionally, acute foot‐shock can promote alcohol‐seeking behaviour reinstatement and facilitate orexin concentrations in brain regions that have been shown to be associated with reward and addiction. Moreover, the inhibition of orexin receptors can attenuate the formation and reinstatement of alcohol addiction‐like behaviour among adolescent mice. Collectively, our findings indicate that orexin system may be a pivotal target for preventing stress‐induced alcohol addiction and reinstatement among the adolescents.

## Graphical abstract

Chronic foot stress increases the vulnerability of adolescent mice to alcohol addiction‐related behaviours. Acute foot stress promotes the recovery of alcohol‐seeking behaviour in adolescent mice and increases orexin concentrations in the hippocampus, nucleus accumbens and prefrontal cortex. Orexin receptor antagonism attenuates the formation and recovery of alcohol‐like behaviour in adolescent mice.

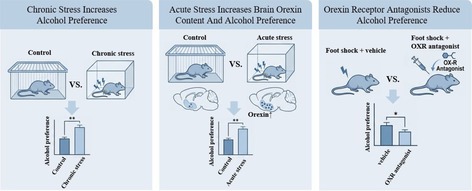

## 1 Introduction

The 2018 report from the World Health Organization (WHO) states that drinking at an early age is a common phenomenon globally, with the prevalence of drinking among 15‐year‐olds ranging from 50% to 70% in many countries, and that more than a quarter of young people between the ages of 15 and 19, totalling approximately 155 million, are alcohol drinkers [1]. Underage drinking is also common in China. In 2022, a targeted quantitative study of secondary school students in nine provinces and cities in China revealed that 42.73% of secondary school students had consumed alcohol [2]. Alcohol exposure during adolescence can have short‐ and long‐term harmful effects, including cognitive and neurological deficits, poor academic performance and mental health problems [3].

The causes of underage drinking behaviour are multifactorial. Among them, stressful events have been shown to be an important contributor to adolescent drinking problems [4]. This is because adolescents are subjected to academic, interpersonal, family and social pressures; these distinctive features of adolescence increase the likelihood of engaging in risky behaviours such as drinking [5]. Studies have shown that stress could be an important risk factor for the initiation and reinstatement of alcohol dependence [6]. Additionally, some clinical studies have revealed that exposure to stressful events can increase alcohol consumption in both alcohol‐dependent and nonalcohol‐dependent people [7]. Similar findings have been reported in animal studies. For example, acute exposure to predator odour stress and acute restraint stress can promote alcohol preference as well as alcohol reinstatement [8]. However, there are also inconsistent reports. One study showed that a single acute exposure to social defeat stress increased alcohol consumption, whereas repeated exposure to the same stress did not [9]. In addition, restraint stressors sometimes fail to promote the reinstatement of alcohol‐seeking behaviour [10]. The related neural mechanisms and the relationships between stress and alcohol dependence have not been fully elucidated, especially among adolescents. Further exploration of this relationship and its underlying mechanisms is important for the prevention and treatment of underage alcohol dependence among adolescents.

Several studies have shown that there is a close relationship between the orexin system and stress. Orexin, which is divided into orexin A and orexin B, is a peptide synthesized and secreted specifically by the lateral hypothalamus (LH), can project to all regions of the brain [11], and can project to all regions of the brain [12]. For example, orexin neurons modulate responses to stressors by projecting to regions such as the locus coeruleus [13]; it can also project to the paraventricular nucleus of the hypothalamus, prompting it to express adrenocorticotropic hormone neurons and activate the hypothalamic–pituitary–adrenal (HPA) axis, thereby enhancing the ability to adapt to severe or chronic stress [14]. Furthermore, several studies suggest that the hypothalamic orexin system also plays key roles in multiple types of addiction‐like behaviours and may be a potential pharmacological target for the treatment of addiction [15]. The orexin system can directly or indirectly innervate dopamine neurons in the bed nucleus of the stria terminalis (BNST), nucleus accumbens (NAc), hippocampus (Hip), the ventral tegmental area (VTA) and so on, suggesting that the orexin system may be involved in substance‐reinforcing effects [16]. For example, orexin 1 receptor (OX1R) antagonists can reduce alcohol consumption and self‐administration [17]. Some studies have indicated that orexins may also be involved in addiction reinstatement [18]. For example, injecting neuropeptide S (NPS) into the hypothalamus, which can promote the release of orexin A by activating orexinergic neurons [19], enhances cue‐induced reinstatement of alcohol preference, and this effect can be diminished by inhibiting OX1R in stress‐related brain regions [19]. These findings provide a significant anatomical basis for the involvement of the orexin system in stress‐induced alcohol dependence reinstatement [19]. Therefore, stress may be an important factor in alcohol addiction and reinstatement, and the orexin system plays a critical role in manipulating the stress response and alcohol addiction.

At present, there are many studies on the relationship between the orexin system and alcohol preference and alcohol‐seeking behaviour reinstatement in animals under stress. The results generally show that blocking orexin receptors can effectively attenuate ethanol‐related place preference formation and reinstatement [20]. However, less attention has been paid to adolescent animals. As mentioned previously, adolescents are in a transitional stage of development, which may increase their vulnerability to alcohol dependence [21]. However, only a few studies have shown that adolescent mice may be more sensitive to stress‐induced alcohol dependence and reinstatement than adult mice [22]. Therefore, more research is needed to elucidate the mechanisms related to stress‐induced addiction and reinstatement during this period, which is important for preventing underage alcohol addiction.

We hypothesized that the orexin system is a significant factor linking stress and adolescent alcohol dependence. To provide a theoretical basis for this hypothesis, we first performed a bioinformatics analysis using publicly available databases (GeneCards, STRING) to construct protein–protein interaction networks among orexin‐, stress‐ and alcohol dependence‐related genes. This analysis confirmed that orexin receptors are topologically connected to key stress and reward pathway genes, supporting their potential role as a molecular hub.

Based on these computational predictions, we employed the conditioned place preference (CPP), the acute/chronic foot‐shock stress models, the Open Field Test (OFT), the Elevated Plus Maze (EPM) and the enzyme‐linked immunosorbent assay (ELISA) to elucidate the role of the orexin system in stress‐induced formation and reinstatement of alcohol addiction‐like behaviour among adolescent mice. For orexin measurement, we focused on the NAc, VTA, mPFC and Hip—major projection targets of orexin neurons that directly modulate reward, stress and memory processes. These regions were selected because orexin concentrations in projection sites reflect neurotransmitter release at behaviourally relevant effector sites, in contrast to the LH which reflects synthesis and storage, or the BNST which primarily integrates stress signals.

## 2 Materials and Methods

### 2.1 Data Collection and Organization

The genes related to orexin, acute stress, chronic stress and alcohol dependence were retrieved in the Genecards database (https://www.Genecards.org/), and the top 25 genes were retained for each retrieval. Gene datasets for orexin, acute stress, alcohol dependence, orexin, chronic stress and alcohol dependence were constructed, and duplicate genes were removed.

### 2.2 Construction and Visualization of PPI Networks

The two gene datasets obtained above were imported into the STRING database (https://string‐db.org/), and the PPI networks related to orexin, acute stress, alcohol dependence and orexin, chronic stress, alcohol dependence were constructed respectively. A screening threshold of at least 0.7 was established, isolated nodes were removed, and the network was optimized to obtain the PPI data of the two networks. Subsequently, the PPI data were imported into Cytoscape3.10.0 for visualization.

### 2.3 Animals

Adolescent male C57BL/6J mice (Hunan SJA Laboratory Animal, China), aged 4 weeks, were housed with same‐treatment littermates (4 per cage). After 1 week of habituation, behavioural testing began at 5 weeks of age. They were subjected to a 12‐h light/12‐h dark cycle, with a temperature of 23°C, and had continuous access to water and food. C57BL/6J mice were selected as study subjects because of their strong responses to stress and their susceptibility to drug abuse and alcohol dependence [23]. All the experimental procedures adhered to animal ethics guidelines and were approved by the Ethics Committee of the Second Xiangya Hospital of Central South University (Approval No. 2020680).

### 2.4 Drugs

Alcohol (20%; v/v) was prepared from 99.9% ethanol (Hu shi SCR, Hunan, China) and 0.9% saline. The mice received an intraperitoneal (i.p.) injection of 20% alcohol in 12.5 mL/kg for a dose of 2 g/kg; many previous studies have shown that this concentration and dose can induce alcohol‐CPP in mice [24]. SB334867, an OX1R antagonist purchased from MCE (MCE Company, UK), was dissolved in 20% (2‐hydroxypropyl)‐β‐cyclodextrin with DMSO as the cosolvent (DMSO volume: 20% (2‐hydroxypropyl)‐β‐cyclodextrin volume = 1:9) and then intraperitoneally injected into the mice in a volume of 10 mL/kg for a dose of 20 mg/kg. The 20 mg/kg dose was selected based on previous studies demonstrating its efficacy in stress‐ and reinstatement‐related paradigms [25, 26], and this dose has no negative effects on eating, grooming or motor activity [27]. JNJ‐10397049, an orexin 2 receptor (OX2R) antagonist purchased from MCE (MCE Company, UK), was dissolved in the same solution as SB334867 and was injected intraperitoneally into the mice in a volume of 10 mL/kg for a dose of 10 mg/kg.

### 2.5 Foot‐Shock Stress

Shock: We subjected mice to foot‐shock stress according to the methods of Nygard et al [28]. The mice were habituated to the apparatus for 5 min before the start of the foot‐shock stress and were then confined to the stress box and received intermittent and uncontrollable foot shocks (intensity: 0.8 mA, duration: 0.5 s, interval: variable, with an average of 40 s and a range of 10–70 s) for 15 min.

### 2.6 Pseudo Shock

The experimental procedure was exactly the same as described above, including auditory cues from the relay, but no real foot shocks were applied.

### 2.7 Elevated Plus Maze Test

EPM was used to assess the anxiety level of mice. Before the test, the mice were transferred to the behavioural laboratory for 30 min to adapt to the environment. Then, the mice were placed at the junction of the open arm and the closed arm (the central area) facing the direction of the open arm, and the mice were allowed to move freely for 5 min [24]. The activity of the mice was recorded using a behavioural tracking system (SMART 2.5, Panlab, Barcelona, Spain). Mice were considered to have visited one arm of the maze if all four limbs left the centre area. We used the percentage of time spent in the open arm (i.e., time in open arm/(time in open arm + time in closed arm), time in open arm %, OT%) to assess anxiety levels, with higher scores indicating lower anxiety levels in mice. The light intensity was 300 lx on the open arms and 100 lx on the closed arms. After each test, 75% alcohol was used to clean the maze.

### 2.8 Open Field Test

The OFT was used to assess the voluntary motor ability and anxiety of the mice. The mice were placed with their backs to the experimenter in the central zone of the clean open field (50% of the total area) and allowed to move freely in the open field for 5 min. A camera system was used to track the movement of the mice. We used the percentage of time spent in the central zone to assess the anxiety level of the mice (time spent in the central zone/total time, time spent in the central zone%). A higher score indicated lower anxiety levels [29]. The brightness remained consistent throughout the procedure. The illumination level in the open field arena was maintained at 100 lx throughout the experiments. After each test, 75% alcohol was used to clean the open field.

### 2.9 Conditioned Place Preference

The CPP apparatus comprised three distinct chambers. An automatic video tracking system (Jiliang Software and Instruments, Shanghai, China) was used to track the movement of mice and the time spent in different chambers. All the mice were placed in the centre chamber with free access to all the compartments for 30 min for 2 days for habituation. We adopted a biased design paradigm [24] because previous studies have indicated that C57BL/6 mice are more likely to develop alcohol‐induced CPP with a biased design [30]. The CPP consisted of the following three phases: acquisition, extinction and stress‐induced reinstatement. The ethanol‐induced CPP was evaluated by comparing the duration spent in the drug‐paired chamber in the pretest, posttest, ext‐test and rei‐test.

#### 2.9.1 Acquisition Phase: Pretest

To assess the initial preferred/nonpreferred chambers, mice were placed in the central chamber with free access to all chambers for 15 min, with the side of the mouse that was active for the longest time being the preferred chamber and vice versa the nonpreferred chamber. Conditioning: Alcohol and saline conditioning sessions were performed alternately in the morning and afternoon for six consecutive days, and the interval of administration was more than 6 h. The interval between morning and afternoon injections (> 6 h) was selected based on previous studies demonstrating that this interval is sufficient for complete ethanol metabolism in C57 mice [31]. The preferred chamber in the pretest was paired with saline and the nonpreferred compartment with alcohol. During the conditioning session, the doors remained closed, and the animals were confined to the drug‐paired chamber or saline‐paired chamber for 45 min. Posttest: After the conditioning session, the mice were allowed free access to all the chambers for 15 min.

#### 2.9.2 Extinction Phase: Extinction Training

Mice that spent 10% more time in the drug‐paired chamber in the posttest than in the pretest continued to the extinction phase; mice that did not meet this criterion were excluded from subsequent phases. The extinction training sessions involved the same procedure as the conditioning phase, except that saline was injected in the morning and afternoon for eight consecutive days. Ext‐test: After the extinction training, the mice were allowed free access to all the chambers for 15 min.

#### 2.9.3 Reinstatement Phase

If the time spent in the paired compartment in the ext‐test was > 10% less than that in the posttest, and there was no significant difference between the ext‐test and pretest, the recovery phase was continued; mice that did not meet this criterion were excluded from subsequent phases. To promote reinstatement of the alcohol‐induced CPP, the mice were exposed to a single foot–shock stress. Rei‐test: After shock stress, mice were allowed free access to all chambers for 15 min.

### 2.10 Locomotor Activity

The automatic monitoring system of the CPP device was used to analyse the total distance travelled by the mice inside the device, which was defined as the locomotor activity. In this study, the total distance the mice moved within the CPP device was recorded during the reinstatement test to determine whether the antagonist affected motor activity [32].

### 2.11 Enzyme‐Linked Immunosorbent Assay

The levels of orexin in the NAc, Hip, mPFC and VTA were determined via ELISA. Studies have shown that these areas are not only projection areas for hypothalamic orexin neurons [12], but may also be involved in stress and reward processing [16]. The mice were injected intraperitoneally with 5% chloral hydrate (0.5 g/kg) for anaesthesia and then decapitated. According to the locations provided in the mouse brain atlas, the NAc, Hip, mPFC and VTA brain regions were separated on ice, weighed and stored at −80°C. The tissue was rinsed with PBS (1×) and chopped into a homogenization tube. The tissue was fully ground in PBS (1×) homogenate with a grinding machine, and the final concentration was 100 mg/mL; the mixture was incubated at −20°C overnight and then frozen and thawed twice to disrupt the cell membrane. The homogenate was subsequently centrifuged at 5000 × g and 4°C for 5 min. The supernatant was retained for ELISA detection. A Mouse Orexin A ELISA Kit (CUSABIO, China) was used for ELISA.

### 2.12 Effect of Chronic Stress on Alcohol‐Induced CPP Acquisition in Adolescent Mice

In Experiment 1, the mice were randomly divided into a chronic stress group (*n* = 7) and a control group (*n* = 7), which received foot shock (15 min/day) and pseudo shock, respectively, for 10 days (Days 1–10). After the 10‐day foot‐shock session, the mice were subjected to the EPM test, OFT, pretest of CPP (Day 11) and alcohol‐induced CPP conditioning (Days 12–17). Finally, a posttest was performed to compare the alcohol‐induced CPP scores (CPP scores were calculated by subtracting the baseline CPP value from their residence time on the drug‐paired side after CPP training) of the two groups (Day 18).

### 2.13 Effects of Orexin Receptor Antagonists on Alcohol‐Induced CPP Acquisition After Chronic Stress

In Experiment 2, all the mice were subjected to foot shock for 10 days before CPP conditioning (Days 1–10) and then received CPP pretest (Day 11). The mice were then randomly divided into SB, JN and vehicle groups, which were preinjected with an OX1R antagonist (SB334867), an OX2R antagonist (JNJ‐10397049) and vehicle (DMSO volume: 20% (2‐hydroxypropyl)‐β‐cyclodextrin volume = 1:9 mixture), respectively, 1 h before each alcohol conditioning session (Days 12–17). The rationale for administering the antagonists prior to every conditioning session was to achieve sustained orexin receptor blockade throughout the entire acquisition phase of CPP, thereby assessing whether the orexin system is required for the formation of chronic stress‐induced alcohol preference. The posttest was subsequently performed (Day 18). By comparing the alcohol‐induced CPP scores of the three groups, the effects of orexin antagonists on chronic stress‐associated alcohol‐induced CPP acquisition were evaluated.

### 2.14 Effects of Acute Stress on Alcohol‐Induced CPP Reinstatement and Orexin Levels in Adolescent Mice

In Experiment 3, after the acquisition (Days 1–8) and extinction (Days 9–17) of alcohol‐induced CPP, the mice were randomly assigned to the stress group or the control group, which received a single foot shock or a pseudo shock, respectively. The rei‐test was subsequently performed immediately, and the mice were sacrificed and sampled within 2 h. The concentration of orexin in different brain regions was determined by ELISA (Day 18).

### 2.15 Effects of Orexin Receptor Antagonists on Alcohol‐Induced CPP Reinstatement After Exposure to Acute Stress in Adolescent Mice

In Experiment 4, after the acquisition (Days 1–8) and extinction (Days 9–17) of alcohol‐induced CPP, the mice were randomly assigned to the SB, JN and vehicle groups, which were injected with JNJ‐10397049, SB334867 and vehicle, respectively, 45 min before receiving a single foot shock. In contrast to the repeated dosing regimen in Experiment 2, a single dose was administered here because we aimed to block orexin signalling specifically at the time of acute stress exposure and reinstatement testing. This design tests whether acute orexin receptor activation at the moment of stress is necessary for the expression of stress‐induced reinstatement, rather than during the acquisition or consolidation phases. After the foot shock, the mice were returned to the CPP apparatus to determine their CPP reinstatement scores (Day 18).

### 2.16 Statistical Analysis

All statistical analyses were performed with SPSS 26.0 software. The Shapiro–Wilk test (S–W test) was used to test for normality, and all values are expressed as the mean ± SEM. A repeated‐measures two‐way analysis of variance (two‐way ANOVA) was used to evaluate the differences between different groups and different stages (treatment factor × stage factor). When there was a cross‐effect between two factors, the Bonferroni multiple comparison method was used for simple effect analysis. An independent samples *t*‐test was used to evaluate the differences between two groups. One‐way analysis of variance (ANOVA) was used to evaluate the differences among three groups. A *p* value of less than 0.05 was considered to indicate statistical significance.

## 3 Result

### 3.1 Screening Results of Genes Related to Orexin, Chronic Stress, Acute Stress and Alcohol Dependence

We retrieved 337 orexin‐related genes, 13 310 chronic stress‐related genes, 16 249 acute stress‐related genes and 11 867 alcohol dependent‐related genes from the Genecards database (https://www.Genecards.org/). After the retrieval, only the top 25 genes in each category were retained. We obtained 71 genes after combining and de‐duplication 75 genes related to orexin, chronic stress and alcohol dependence. Similarly, the combination of the 75 genes for orexin, acute stress and alcohol dependence yielded 71 genes.

### 3.2 Construction of Protein–Protein Interaction Networks

**FIGURE 1:**
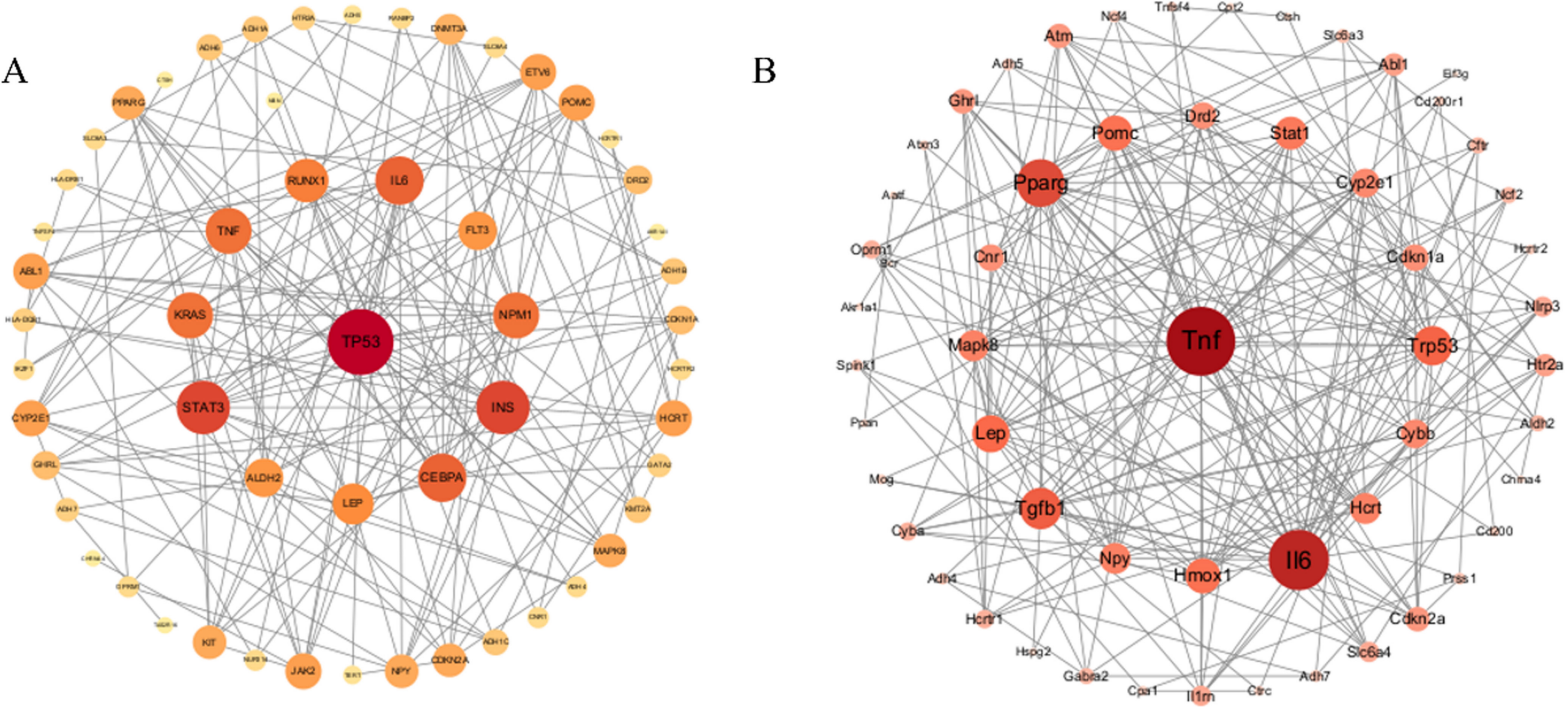
The relationship between the orexin system and acute stress and alcohol dependence. (A) The PPI network of orexin system, chronic stress and alcohol‐dependent genes, with each node representing a protein and each edge representing a protein–protein interaction relationship; nodes with darker colour and larger area have higher degree. (B) The PPI network of orexin system, acute stress and alcohol‐dependent genes, with each node representing a protein and each edge representing a protein–protein interaction relationship; nodes with darker colour and larger area have higher degree.

PPI analysis showed that the orexin system, chronic stress‐related genes and alcohol dependence‐related genes formed an interconnected network consisting of 56 nodes and 228 edges (Figure 1A). In the same way, PPI analysis also showed that the orexin system, acute stress‐related genes and alcohol dependence‐related genes formed an interconnected network consisting of 55 nodes and 183 edges (Figure 1B). These networks indicate that the orexin system is molecularly connected to both stress and alcohol dependence pathways, providing a theoretical basis for our experimental hypothesis.

### 3.3 Effects of Chronic Stress Exposure on the Acquisition of Alcohol‐Induced CPP in Adolescent Mice

**FIGURE 2:**
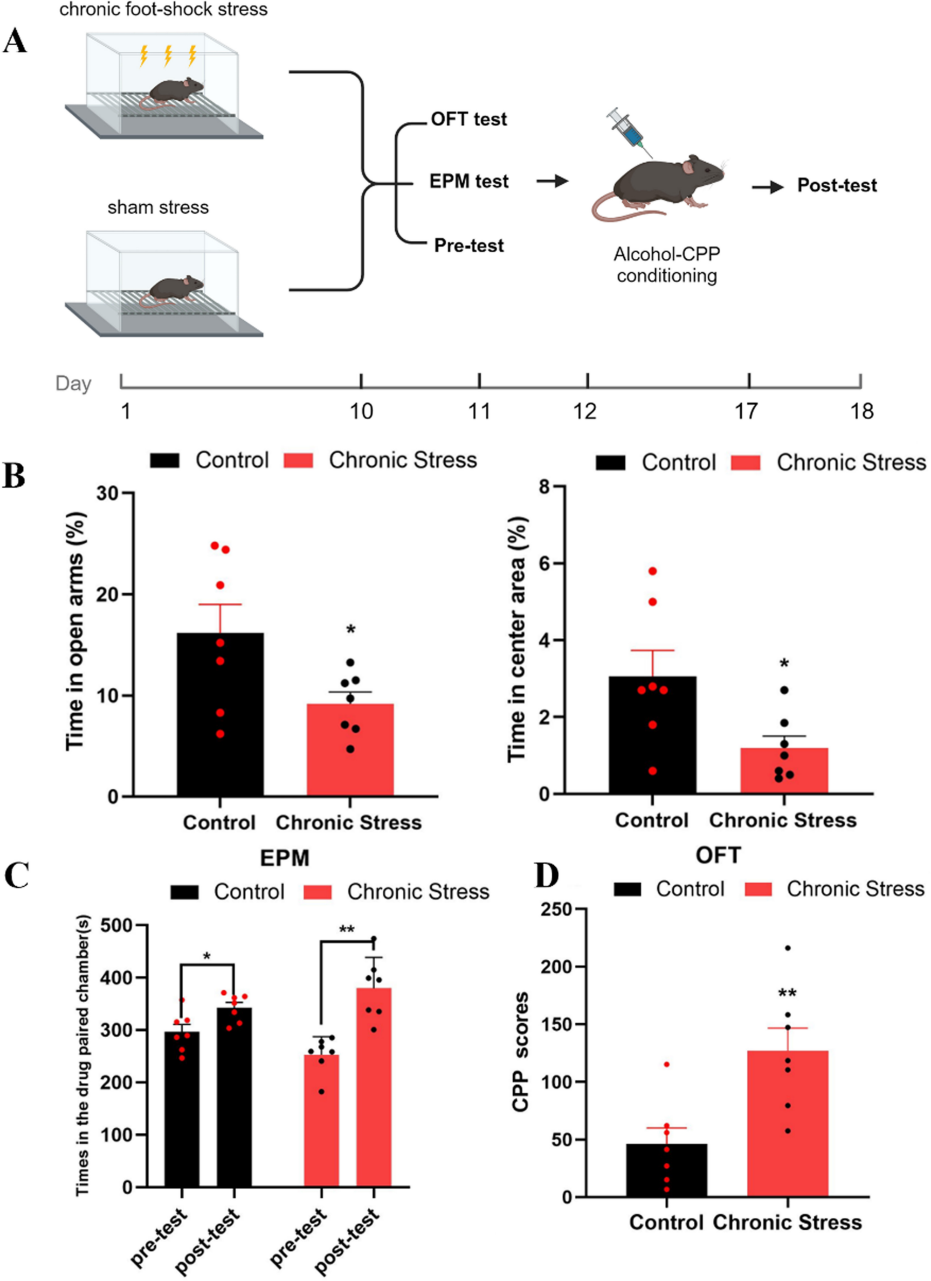
Chronic foot‐shock stress increased alcohol‐induced CPP scores in adolescent male mice. (A) Schematic diagram of the timeline for Experiment 1. Illustration from www.biorender.com. (B) Left: The chronic stress group (red, *n* = 7) spent significantly less time in the open arm in the EPM test than did the control group (black, *n* = 7) (independent samples *t*‐test, *p < 0.05, 9.68% ± 1.13% vs. 16.17 ± 2.82%, df = 12). Right: The chronic stress group (red, *n* = 7) spent significantly less time in the central area in the OFT than did the control group (black, *n* = 7) (independent samples *t*‐test, *p < 0.05, 1.28 ± 0.28% vs. 3.06 ± 1.79%, df = 12), data shown as the mean ± SEM.(C) After alcohol‐induced CPP training, the duration of time spent in the alcohol‐paired chamber significantly increased in both the control group (blue, *n* = 7, paired *t*‐test, *p < 0.05, df = 6) and the chronic stress group (red, *n* = 7, paired *t*‐test, **p < 0.01, df = 6). The data are shown as the mean ± SEM. (D) The chronic stress group (red, *n* = 7) had significantly higher ethanol‐induced CPP scores than did the control group (blue, *n* = 7) (independent samples *t*‐test, **p < 0.01, 126.79 ± 19.98 vs. 46.17 ± 13.81, df = 12); the data are presented as the mean ± SEM, total *n* = 14.

In Experiment 1, the chronic stress group spent a significantly lower percentage of time in the open arm during the EPM test than the control group (*p < 0.05), and also spent a significantly lower percentage of time in the central area during OFT than the control group (*p < 0.05), as detailed in Figure 2B. The results after CPP training showed that the chronic stress group (**p < 0.01) and the control group (*p < 0.05) spent significantly more time in the alcohol‐paired chamber than in the other paired chamber (see Figure 2C). Although there was no significant difference in posttest results between the two groups (*p* > 0.05), the CPP scores in the chronic stress group were approximately twice as high as those in the control group (**p < 0.01), as shown in Figure 2D. Thus, chronic foot‐shock stress significantly increased susceptibility to alcohol preference in adolescent mice.

### 3.4 Effects of Orexin Receptor Antagonists on the Acquisition of Alcohol‐Induced CPP After Chronic Foot‐Shock Stress in Adolescent Mice

**FIGURE 3:**
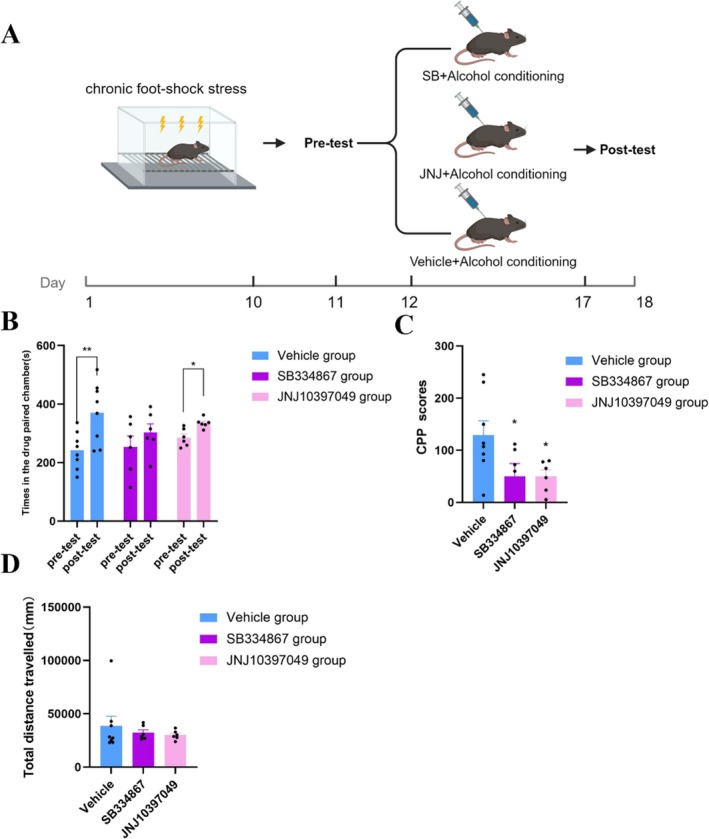
OX1R and OX2R antagonists suppressed chronic stress‐associated alcohol‐induced CPP acquisition in adolescent mice. (A) Schematic diagram of the timeline for Experiment 2. Illustration from www.biorender.com. (B) The vehicle group (blue, *n* = 7, paired *t*‐test, **p < 0.01, 241.77 ± 22.35 vs. 371.00 ± 36.71, df = 6) and the SB group (purple, *n* = 7, paired *t*‐test, *p < 0.05, 335.22 ± 6.81 vs. 285.3 ± 12.51, df = 6) spent more time in the drug‐paired chamber after alcohol‐induced CPP training, whereas the JN group did not (red, *n* = 7, paired *t*‐test, *p* > 0.05, 303.52 ± 29.42 vs. 253.63 ± 37.88, df = 6), data shown as the mean ± SEM. (C) The vehicle group (blue, *n* = 7) had significantly higher alcohol‐induced CPP scores than did the SB (purple, *n* = 7) and JN groups (red, *n* = 7) (one‐way ANOVA, *p < 0.05, 49.88 ± 24.82 or 49.92 ± 12.38 vs. 129.22 ± 27.37, df = 20), data shown as the mean ± SEM, total *n* = 21. (D) No significant difference in total distance travelled was observed among the three groups of mice (one‐way ANOVA, *p* > 0.05, df = 20; data are shown as the mean ± SEM, total *n* = 21).

In Experiment 2, a preference for alcohol‐paired chambers was formed in the vehicle group and JN group (*p < 0.05) but not in the SB group (*p* > 0.05), as detailed in Figure 3B. In addition, CPP scores were significantly lower in both SB and JN groups than in the vehicle group (*p < 0.05), although the JN group still exhibited a significant preference for the alcohol‐paired chamber, as detailed in Figure 3B,C. In addition, there was no significant difference in motor activity among the three groups (*p* > 0.05), as detailed in Figure 3D. These findings indicated that OX1R antagonism completely blocked, whereas OX2R antagonism partially attenuated the enhanced vulnerability to chronic stress‐induced alcohol dependence and showed that this effect was not caused by a reduction in motor activity.

### 3.5 Effects of a Single Acute Stress Exposure on Alcohol‐Induced CPP Reinstatement in Adolescent Mice

**FIGURE 4:**
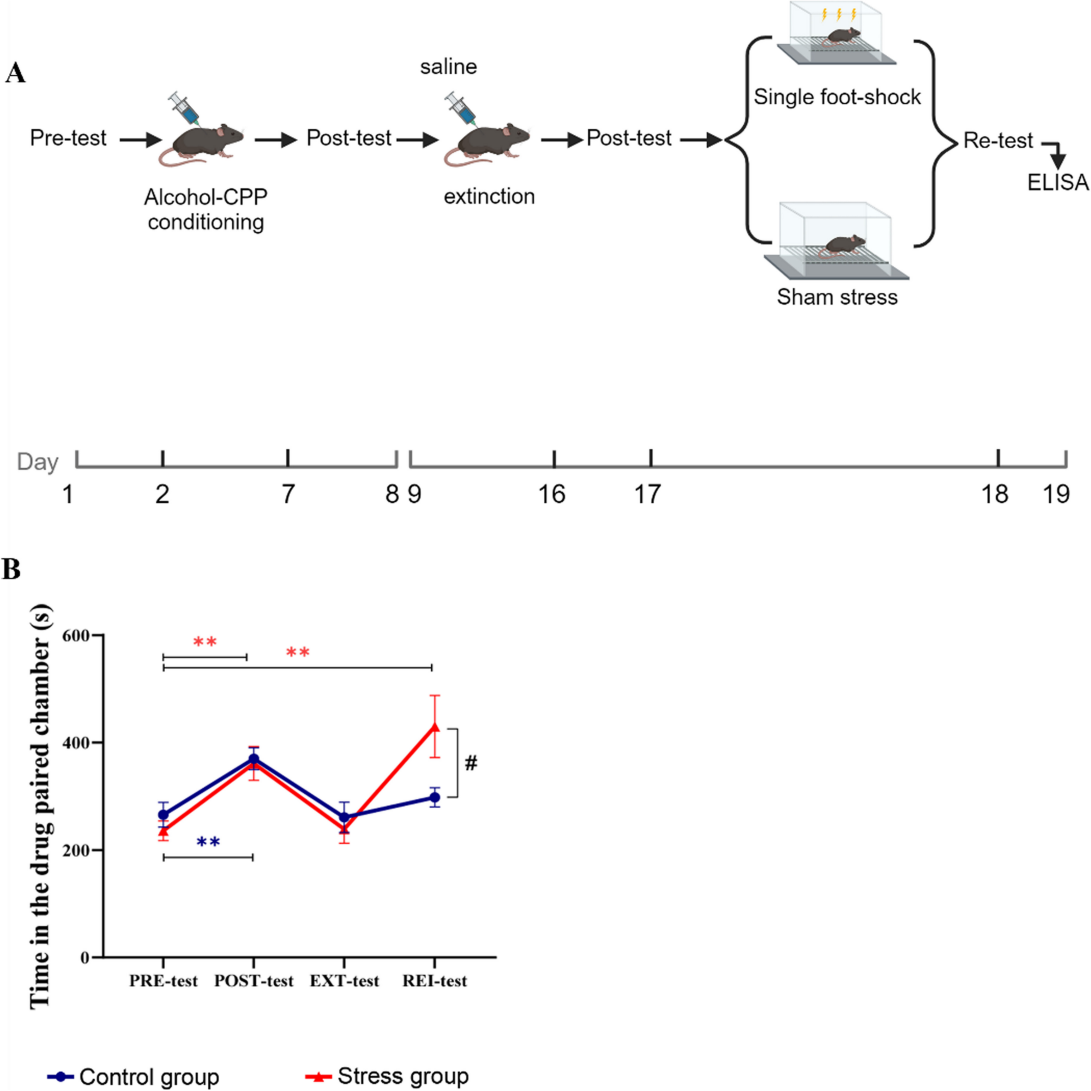
Acute foot shock facilitated the reinstatement of alcohol‐induced CPP. (A) Schematic diagram of the timeline for Experiment 3. Illustration from www.biorender.com. (B) Two‐way ANOVA showing the interaction effect of test group (*F* = 3.489, *p < 0.05, df = 3). The residence time of both the control group (blue, *n* = 6) and stress group (red, *n* = 8) in the alcohol‐paired chamber in the posttest were significantly greater than that in the other chamber (Bonferroni multiple comparisons, simple effects test **p < 0.01, df = 1). There was no difference in residence time in the alcohol‐paired chamber between the control and stress groups in the ext‐test or the pretest (Bonferroni multiple comparisons, simple effect of test: *p* > 0.05, df = 1). However, a single acute foot shock before alcohol injection significantly increased the CPP score in the rei‐test in the stress group but not in the control group (Bonferroni multiple comparisons, simple effect test: **p < 0.01, simple effect of group: *p < 0.05, df = 1). The data are presented as the mean ± SEM, total *n* = 14.

In Experiment 3, two‐way (test factor × group factor) repeated‐measures ANOVA was conducted and revealed a significant interaction effect between the two factors (*F* = 3.489, *p* = 0.048). The Bonferroni multiple comparison method was subsequently employed to conduct simple effect analysis. As shown in Figure 4B, the alcohol‐induced CPP acquisition (**p < 0.01) and extinction (*p* > 0.05) models were successfully established in both the control and stress groups. However, when the residence time was compared between the rei‐test and pretest stages, *P* was < 0.01 in the stress group and *P* was > 0.05 in the control group [8]. Furthermore, the residence time during the rei‐test stage was significantly longer in the stress group than in the control group (*p < 0.05). After the extinction of alcohol‐induced CPP in adolescent mice, successful CPP reinstatement was observed only in the stress group (**p < 0.01), which experienced acute foot‐shock stress.

### 3.6 Changes in Orexin Concentrations in Specific Brain Regions After Acute Stress

**FIGURE 5:**
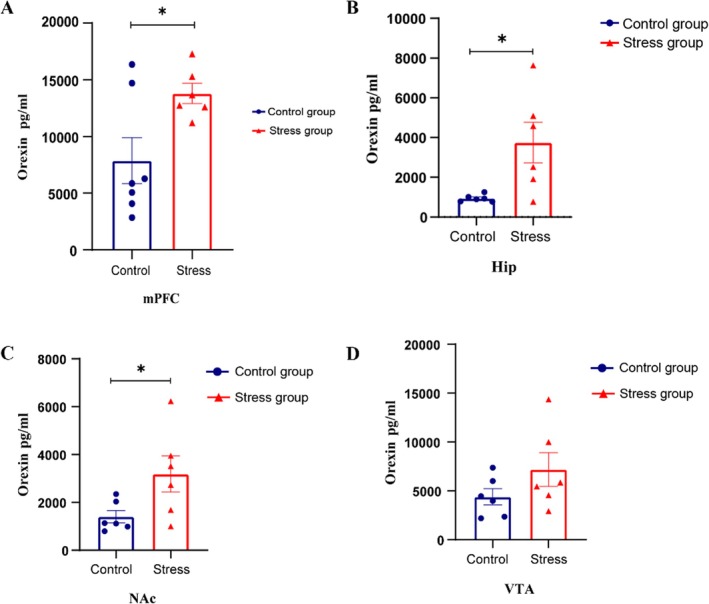
Acute foot shock promoted orexin expression in the mPFC, Hip and NAc. (A) The orexin level in the mPFC in the stress group (red, *n* = 8) was significantly greater than that in the control group (blue, *n* = 6) (independent samples *t*‐test, *p < 0.05, 13801.58 ± 887.93 vs. 7878.94 ± 2029.10, df = 12). (B) The orexin level in the Hip in the stress group (red, *n* = 8) was significantly greater than that in the control group (blue, *n* = 6) (independent samples *t*‐test, *p < 0.05, 3748.51 ± 1021.21 vs. 941.16 ± 72.87, df = 12). (C) The orexin level in the nucleus NAc in the stress group (red, *n* = 8) was significantly greater than that in the control group (blue, *n* = 6) (independent samples *t*‐test, *p < 0.05, 3186.35 ± 757.05 vs. 1402.87 ± 257.50, df = 12). (D) The orexin level in the VTA region in the stress group (red, *n* = 8) tended to be greater than that in the control group (blue, *n* = 6) (independent samples *t*‐test, *p* > 0.05, 7189.44 ± 1723.91 vs. 4392.49 ± 829.78, df = 12). The data are presented as the mean ± SEM, total *n* = 14.

Orexin concentrations in the mPFC, hip and NAC were significantly increased in the stress group compared with the control group (*p < 0.05), as detailed in Figure 5A–C. In addition, compared with the control group, the concentration of orexin in the VTA of the stress group had a tendency to increase, but it was not significant (*p* > 0.05) (see Figure 5D for details). The different changes in orexin concentrations in different brain regions may indicate that the orexin system modulates specific behaviours in particular brain regions. Combined with our results, the orexin system in the NAc, Hip and mPFC regions could be strongly associated with stress processing. These findings require further exploration.

### 3.7 Effects of Orexin Receptor Antagonists on Alcohol‐Induced CPP Reinstatement After Exposure to Acute Stress in Adolescent Mice

**FIGURE 6:**
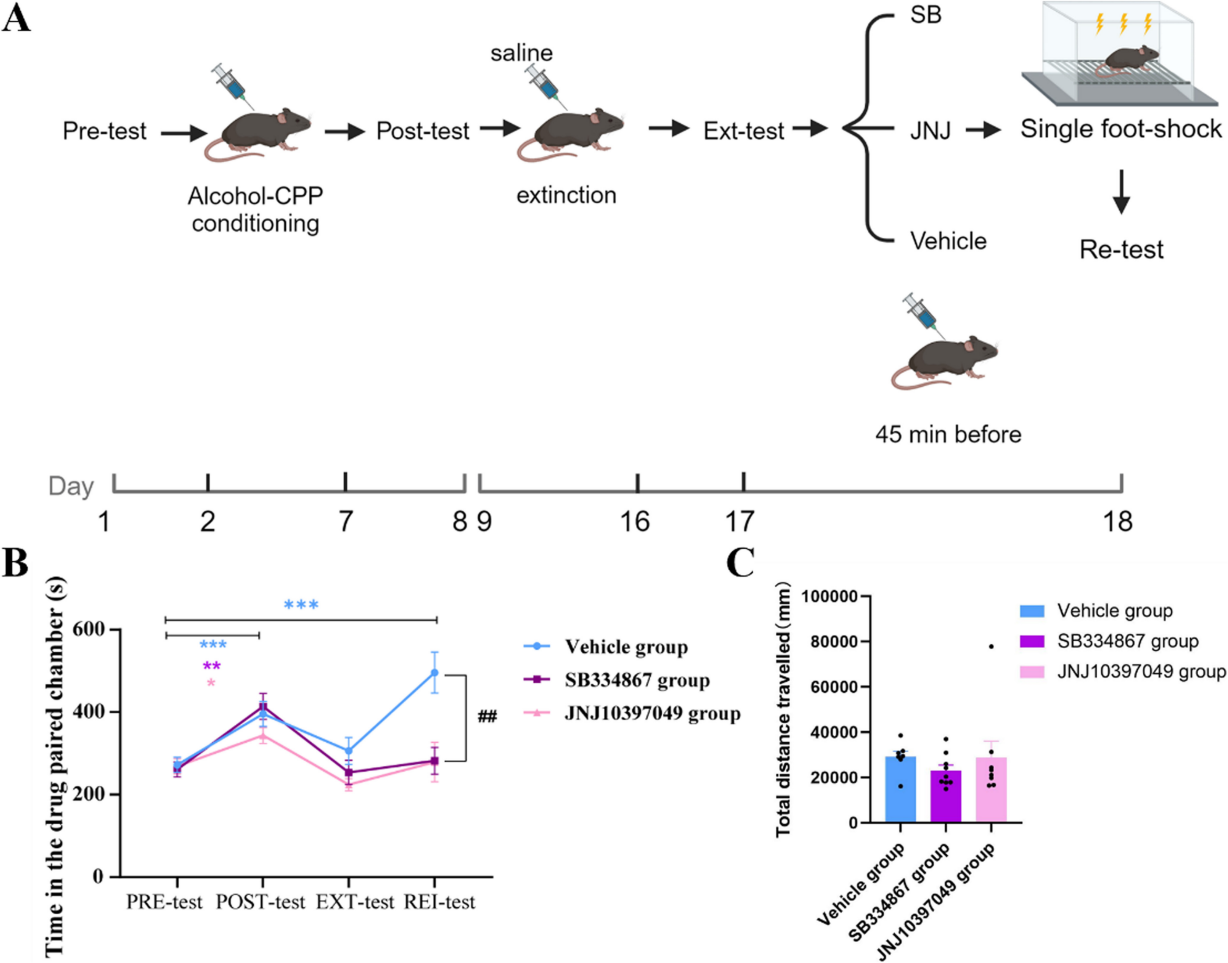
Intervention with orexin receptor antagonists could block alcohol‐induced CPP reinstatement induced by a single foot shock. (A) Schematic diagram of the timeline for Experiment 4. Illustration from www.biorender.com. (B) Two‐way ANOVA showing the interaction effect of test group (*F* = 5.405, *** *p* < 0.001, df = 3). The time spent in the drug‐paired chamber in the posttest in the vehicle group (blue, *n* = 7), the SB group (purple, *n* = 8), and the JN group (red, *n* = 9) was significantly greater than that spent in the other chamber (Bonferroni multiple comparisons, simple effects test: *p < 0.05, **p < 0.01, ***p < 0.001, df = 3). There was no difference in residence time in the drug‐paired chamber among the three groups in the ext‐test or pretest (Bonferroni multiple comparisons, simple effect of test: *p* > 0.05, df = 3). However, the residence time in the drug‐paired chamber in the vehicle group significantly increased in the rei‐test compared with the pretest (Bonferroni multiple comparisons, simple effect of test: ***p < 0.001, df = 3), whereas the SB group and the JN group did not show this pattern (Bonferroni multiple comparisons, simple effect of test *p* > 0.05, df = 3). Compared with the SB group and the JN group, the vehicle group had a greater residence time in the drug‐paired chamber in the rei‐test (Bonferroni multiple comparisons, simple effect of group: **p < 0.01, df = 2). The data are presented as the mean ± SEM, total *n* = 24. (C) There was no significant difference in total distance travelled among the three groups of mice (one‐way ANOVA, *F* = 0.58, *p* > 0.05, df = 23; data are shown as the mean ± SEM, total *n* = 24).

In Experiment 4, as shown in Figure 6B, two‐way ANOVA revealed that all three groups of mice established alcohol‐induced CPP (interaction effect of test group: ***p < 0.001, simple effect of group: *p < 0.05) and successfully underwent extinction (simple effect of group: *p* > 0.05). However, after a single foot shock, the residence time in the alcohol‐paired chamber in the rei‐test in the vehicle group significantly increased compared with that in the pretest (simple effect of group: ***p < 0.001), whereas the SB and JN groups did not show this pattern (simple effect of group: *p* > 0.05). Additionally, the time spent in the alcohol‐paired chamber in the vehicle group in the rei‐test was significantly greater than that in the SB and JN groups (simple effect of test: **p < 0.01), indicating that both OX1R and OX2R receptor antagonists could prevent alcohol‐induced CPP reinstatement induced by acute stress in adolescent mice [33, 34]. In addition, during the rei‐test, the total distance travelled by the mice was recorded to assess locomotor activity. The results, as depicted in Figure 6C, revealed no significant differences among the three groups (one‐way ANOVA, *p* > 0.05).

## 4 Discussion

Our bioinformatics analysis, conducted prior to the animal experiments, predicted that orexin receptors are embedded within a protein–protein interaction network connecting stress‐ and reward‐related genes. This computational framework supports the hypothesis that orexin receptors may serve as a molecular hub integrating stress and reward signals, providing a rationale for our pharmacological interventions.

In conclusion, the orexin system may influence the formation and reinstatement of alcohol dependence under chronic and acute stress. Studies have shown that stress and adversity in early life increase susceptibility to addiction formation, maintenance and reinstatement [35]. Most existing studies have been conducted on adult mice, indicating that stress is an important factor in promoting alcohol craving, self‐administration and alcohol‐induced behavioural sensitization [9]. For example, the studies by Bahi and Macedo et al. found a significant increase in alcohol consumption in adult mice after foot shock [36, 37]. However, adolescents are more vulnerable to the effects of drugs such as alcohol because their brains are not fully developed, especially in the prefrontal cortex, which is responsible for decision‐making and impulse control [38]. Therefore, this study was validated using 4‐week‐old mice. We found that chronic stress could lead to higher anxiety levels and alcohol‐induced CPP in adolescent mice, suggesting that chronic stress might be a contributing factor to the formation of alcohol dependence in adolescents. Studies have shown that orexin receptors may be important regulators of reward, motivation and functional interactions between orexin and dopamine neurons in the mesolimbic system and are involved in the rewarding effects of substances [39]. Francisco et al. showed that orexin receptor antagonists prevented the reinstatement of alcohol dependence in adult rats after alcohol withdrawal under stress [40]. Gabriel et al. also demonstrated that orexin receptor antagonists significantly reduced alcohol consumption in alcohol‐dependent rats [41]. Therefore, we injected both OX1R and OX2R antagonists into adolescent mice and provided new evidence that both OX1R and OX2R antagonists attenuate the acquisition and reinstatement of alcohol‐induced CPP in adolescent mice without impairing locomotor activity.

Clinical studies have shown that stress can affect all stages of addiction, especially the withdrawal phase [42], which can lead to reinstatement of alcohol dependence [43]. Our research verified the above view. After the regression of alcohol‐induced CPP, exposure to acute stress can significantly promote the recovery of alcohol‐induced CPP in adolescent mice, suggesting that acute stress may be a key factor in the reinstatement of alcohol‐seeking behaviour in adolescents. This is consistent with the research results of Bhutada et al., who found that giving a single restraint stress or forced swimming stress to adult mice in the withdrawal period within 10 min could induce their alcohol‐seeking behaviour [8]. According to a previous review, stress exposure during the late extinction stage could consolidate extinction memory and lower the probability of reinstatement, whereas stress exposure immediately prior to the reinstatement stage could damage extinction memory and contribute to addiction reinstatement [44]. However, the above conclusions are mainly focused on adult mice, and there are still some gaps in the study of adolescent mice. Our study highlights acute stress as a risk factor for reinstatement of alcohol preference in adolescent mice.

Our research indicated that compared with pseudo stress, adolescent mice exposed to acute stress before alcohol‐induced CPP reinstatement showed significantly higher orexin levels in the mPFC, Hip and NAc, and there was an increasing trend in orexin levels in the VTA. This is consistent with many previous research results. For instance, Georgescu et al. found that foot shock can activate orexin neurons and promote the release of orexin [45]. Li et al. showed that acute restraint can significantly increase orexin levels in the NAC of adult mice [46]. In addition, injecting orexin into the VTA can induce stress‐related drug‐seeking behaviour [47]. The NAc, mPFC, Hip and VTA are all regions involved in reward processing, and they are also associated with the mesolimbic dopaminergic system [48]. And all these brain regions receive projections from orexin neurons. Thus, our results suggest that exposure to acute stress after withdrawal can significantly activate orexin expression in reward‐related regions of the brain in adolescent mice, leading to an imbalance of the orexin system.

Finally, we demonstrated that OX1R antagonists were effective in preventing the reinstatement of alcohol‐seeking behaviour triggered by acute stress. This is consistent with the findings of Martin et al [33]. They found that systemic or intracerebral administration of OX1R antagonists to rats reduced drug‐seeking behaviour induced by alcohol‐related cues. Yohimbine is an alpha‐2 adrenergic receptor agonist that is frequently used to mimic drug‐induced stress and can promote reinstatement of alcohol addiction, but it can be inhibited by OX1R antagonists [47]. Although a preference was still formed in the JNJ group in our study, existing studies have shown that OX2R antagonists can reduce stress‐induced ACTH release and prevent drug‐seeking behaviour [49] However, in our study, we did not inject orexin receptor antagonists into specific brain regions, thus failing to accurately investigate orexin receptor function. In the future, we need to clarify the role of orexin receptors in specific brain regions that are involved in stress‐induced reinstatement of alcohol dependence. While most of the above studies have used adult animal models, our study focused on adolescent models and incorporated partial bioinformatics approaches, providing evidence that OX1R and OX2R antagonists may modulate acute stress‐induced reinstatement of alcohol dependence in adolescents. However, only male mice were used, limiting generalizability to females. This is particularly relevant given that adolescence is characterized by profound sex‐specific changes in sex steroid hormones that can influence both stress processing and alcohol reward. Otherwise, we did not measure circulating hormone levels. Future studies incorporating both sexes are needed to fully understand the interplay between pubertal hormones, orexin signalling and stress‐induced alcohol dependence. Finally, this study used forced ethanol administration (intraperitoneal injection), which differs from human voluntary drinking. Future studies employing voluntary drinking models (e.g., two‐bottle choice and operant self‐administration) are needed to extend our findings.

## 5 Conclusions

In conclusion, the present study suggests that orexin, chronic/acute stress and alcohol dependence are not isolated systems, and there is a clear interaction between them. Chronic stress increased anxiety in adolescent mice, which made them more prone to alcohol addiction behaviour. This effect could be significantly attenuated by OX1R and OX2R antagonists. In addition, acute stress caused the reinstatement of alcohol‐seeking behaviour in adolescent mice and increased orexin concentration in reward‐related brain regions. Inhibition of OX1R or OX2R could alleviate the reinstatement of alcohol‐seeking behaviour induced by acute stress. Based on these findings, OX1R and OX2R may be targets for the treatment of stress‐induced alcohol addiction and reinstatement in adolescents. Elucidating the relationship between the stress system, orexin system and alcohol dependence may help to explore some of the neurobiological mechanisms of stress‐related alcohol dependence and reinstatement in adolescents.

## Author Contributions

**Wenhao He:** writing – original draft, conceptualization, investigation, methodology, data analysis. **Tianshu Zhao:** writing – original draft, writing – review and editing, investigation, visualization. **Xiaojun Xiang:** conceptualization, writing – review and editing, data curation, supervision. **Li Deng:** writing – review and editing, data curation. **Huilin Wu:** writing – review and editing, data curation.

## Funding

This work was supported by the Natural Science Foundation of Hunan (Grant No. 2021JI30962), and the National Natural Science Foundation of China (Grant No. 82471519).

## Ethics Statement

The Animal Care and Use Committee of Second Xiangya Hospital of Central South University (Hunan, China) reviewed and approved the animal experimental protocols and the treatment procedures.

## Consent

All authors agreed with the content and all gave explicit consent to submit.

## Conflicts of Interest

The authors declare no conflicts of interest.

## Data Availability

The datasets generated during and/or analyzed during the current study are available from the corresponding author on reasonable request.
